# Grit, discounting, & time inconsistency

**DOI:** 10.1007/s11166-025-09456-8

**Published:** 2025-06-17

**Authors:** Christian König-Kersting, Stefan T. Trautmann

**Affiliations:** 1 Department of Banking and Finance, University of Innsbruck, Universitätsstraße 15, 6020 Innsbruck, Austria; 2 Chair of Behavioral Finance, Institute for Economics, University of Heidelberg, Alfred-WeberBergheimer Straße 58, 69115 Heidelberg, Germany; 3 Tilburg University, Tilburg, The Netherlands

**Keywords:** Time preferences, Time inconsistency, Decreasing impatience, Grit, Household finance, Health, D15, G51, I10

## Abstract

We study the association of the perseverance of effort and the consistency of interests components of the psychological measure of grit with economic measures of impatience and time inconsistency in the general population. We find that impatience is associated with grit through the perseverance of effort component. No association of time inconsistency with grit is found. Predicting participants’ financial and health outcomes and behaviors, we find that impatience and grit are predictive for both outcomes, but this is not the case for time inconsistency. Our findings suggest that it can be beneficial for empirical studies of intertemporal decisions to include economic impatience and psychological grit measures.

**Supplementary Information:**

The online version contains supplementary material available at 10.1007/s11166-025-09456-8.

## Introduction

The concept of *Grit* has attracted significant attention since it was first introduced by Duckworth et al. (2007). Grit is thought to measure an individual’s perseverance and long-term goal orientation, and consists of two principal components: *Consistency of Interests* (CoI) and *Perseverance of Effort* (PoE) (see e.g., Datu et al., 2016; Duckworth & Quinn, 2009; von Culin et al., 2014). Grit has been studied in a variety of contexts, and has been linked to such diverse topics as intelligence, happiness, educational attainment, health behavior, labor market outcomes, and financial decision-making (cf. Alan et al., 2019; Arco-Tirado et al., 2019; Cornaggia et al., 2020; Datu et al., 2021; Kannangara et al., 2018; Li et al., 2018; Reed, 2014; Rutberg et al., 2020; Zisman & Ganzach, 2020). Grit appears to be increasing in early childhood (Sutter et al., 2022), and it has been suggested that active interventions increasing grit in children may lead to substantial economic benefits for individuals in the long run (Alan et al., 2019; Sutter, 2014). While the majority of studies highlight the positive effects of grit, Alaoui and Fons-Rosen (2021) demonstrate that gritty individuals may fall victim to their own disposition and continue a course of action beyond the point at which they would have—ex-ante—liked to stop.

The psychological concept of perseverance of effort, with current cost incurred for future benefits, is closely related to delayed gratification and patience, respectively impatience, which economists see as a manifestation of discounting, i.e., a measure of time preference. Patience has been shown to affect life outcomes in various areas from schooling choices to reaching old age (Angerer et al., 2023; Norrgren, 2022). Patience varies not just with socio-economic background but also age and appears to be malleable in early life (Epper et al., 2020; Sutter et al., 2015, 2019; Thompson et al., 2020). While the study of time preferences started out by measuring discount rates as constant measures of impatience (Samuelson, 1937), more recent developments acknowledge that discounting and impatience do not have to be constant, but may be increasing or decreasing with time delay (e.g., Strotz, 1955; Loewenstein & Prelec, 1992; Bleichrodt et al., 2009; Rohde, 2019; and for a review: Cohen et al., 2020).^1^ Such non-constant discounting has theoretically been linked to time inconsistent behavior and planning failure, and has been employed to explain a variety of behaviors and outcomes, including poor financial and health outcomes such as under-saving or obesity (e.g., Backes-Gellner et al., 2021; Bradford et al., 2017; Frederick et al., 2002; Meier & Sprenger, 2010; Merkle et al., 2022; Sutter et al., 2013). Time inconsistency has a natural counterpart in the consistency of interest facet of grit. However, despite the apparent mapping of the important economic concepts of discounting and time inconsistency on the two dimensions of the psychological measure of grit, there is no evidence yet on the empirical relationship between these concepts.

There is some literature linking time preferences to conscientiousness (e.g., Rustichini et al., 2016; Daly et al., 2009; Letkiewicz & Fox, 2014; Manning et al., 2014), which has been argued to have considerable overlap with the concept of grit (e.g., Credé et al., 2017; Ponnock et al., 2020; Schmidt et al., 2018). Conscientiousness is a broader construct encompassing traits like organization, responsibility, and impulse control. Grit’s narrower focus on goal pursuit and perseverance distinguishes it from conscientiousness, though empirical studies reveal moderate to high correlations between the two (Credé et al., 2017).^2^ There is also a growing body of literature attempting to connect personality traits to measures of economic preferences. Borghans et al. (2008) provide a foundational review, emphasizing the importance of integrating personality traits into economic models to understand behaviors such as risk aversion, time preference, and social preferences. Jagelka (2024) extends this work by demonstrating a stronger link between individual abilities and preferences than previously established, using factor analysis to map these relationships empirically. Heckman et al. (2019) underline that the combined study of economic preferences and psychological traits accelerates the generation of knowledge and helps to design effective policy measures.

Despite the broad interest in grit, there is little evidence yet on its relevance for economic outcomes in representative populations (Lechner et al., 2019), and no evidence on its relationship with measures of time preference. Given the relevancy of both the economic and psychological measures for policy in several domains of regulation, a deeper understanding of their relationship is warranted. To this end, the current paper presents the first joint measurement of grit, time discounting, and time inconsistency for a large probability sample of the population. Specifically, we measure the Grit-S scale for more than 3000 members of the LISS panel, a representative panel of the Dutch population, and combine it with incentivized measurements of impatience based on Rohde (2019), which also allow us to identify deviations from constant discounting. To probe the external validity of the grit measure, discount rates, and time inconsistency in the two important policy domains of household finance and health, we add two sets of analyses. First, we survey participants regarding their satisfaction with their own financial and health-related decision-making. If our measures of intertemporal preference and behavior capture people’s failure to implement their own long-term goals in dynamic intertemporal settings, we predict that they correlate meaningfully with measures of dissatisfaction with one’s own behavior and outcomes. Second, we make use of existing indicators of personal finances and health, both subjective and objective, available on the LISS panel. Here we test if grit and time preference predict outcomes in the cross section of the population.

Our findings show that the Dutch representative sample is moderately impatient with an average willingness to wait of four months for an additional €15 (on top of €100). This implies an annualized discount rate of 63%. About 43% of participants exhibit constant discounting across the two time horizons we study, with the remaining participants showing decreasing impatience to a larger degree than increasing impatience (33% versus 23%). As hypothesized, impatience appears to be negatively associated with the perseverance of effort component of grit. However, we do not find systematic evidence of deviations from constant discounting correlating with the consistency of interest component of grit. Participants are on average dissatisfied with both their financial and their health-related situation. Dissatisfaction in both domains correlates negatively with grit. Despite its correlation with grit, impatience sustains predictive power for dissatisfaction in the financial domain. Grit is also predictive of broader health and financial outcomes, with again impatience sustaining some predictive power for financial outcomes.

Section 2 lays out the details of our design. Section 3 reports results regarding the relationship between grit and time preference, and Section 4 presents results on the predictive power of these measures for financial and health satisfaction and outcomes. Section 5 discusses the findings in the light of previous research and draws conclusions for future work with these concepts.

## Study design

We conduct a three-part study on the LISS panel. In part 1 we elicit time preferences. Part 2 comprises the elicitation of ambiguity attitudes and is reported on in a companion paper (König-Kersting & Trautmann, 2025). In part 3 we measure participants’ grit and collect self-reports on their satisfaction regarding household finances and health-related behaviors. In the study, either part 1 (time preferences) or part 2 (ambiguity attitudes) is randomly selected for payment. We augment the data collected in our experiments and questionnaires with demographic, financial, and health data available on the LISS panel.

### Time preference elicitation

We elicit time preferences using choice lists, in which participants always choose between two timed payoffs (i.e., delayed payoffs), referred to as Option A and Option B. Throughout a choice list, timing and payoff associated with Option A remain fixed. Option A always involves a payoff of €100. For Option B, we vary the point in time the payment is received, while the payoff itself remains unchanged at €115.

We employ two such choice lists with different up-front delays to be able to identify and quantify increasing and decreasing impatience (Rohde, 2019). In the first choice list, Option A always pays in 5 weeks, while the payoff delay of Option B increases monotonically from 5 to 55 weeks. We call this the *5-week* list. In the second choice list, all delays are increased by an additional 3 weeks. That is, the delay for Option A is 8 weeks, while the delay for Option B ranges from 8 to 58 weeks. We refer to this second list as the *8-week* list.

**Fig. 1:**
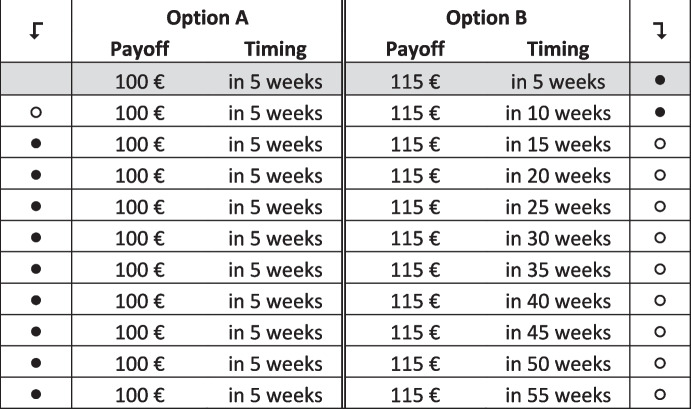
5-week, coarse choice list. This example shows switching after week 10 on the coarse list

**Fig. 2:**
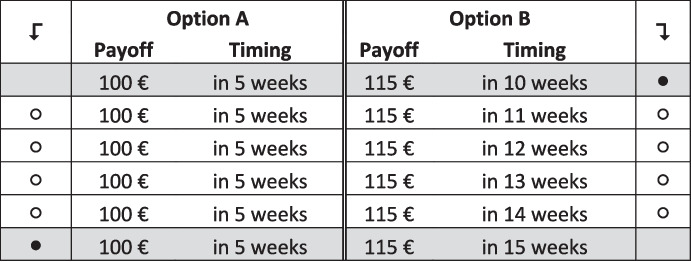
5-week, fine choice list, with switch after 10 weeks. This example shows the zoomed-in choice list based on switching from B to A after week 10 on the coarse choice list (5-week) shown in Fig. 1. The first and the last line correspond to the lines selected in the coarse choice list and were disabled, such that participants could only refine, but not change their switching point

To keep the choice lists shorter and increase data quality, we employ an iterative method and enforce a single switching point per list. We first present participants with a choice list with time steps *Δt* between options equal to 5 weeks (‘the coarse list’, see Fig. 1). Implementing a maximum time difference of 50 weeks, the first choice list comprises 11 items. For each item, participants have to choose between Options A and B; consistency dictates the participants start with Option B and switch at most once to Option A and then stick with this option until they reach the bottom of the list. Thus, we have participants choose the longest delay for which they are willing to choose the larger payoff Option B, and have all other choices automatically filled in. Participants can adjust the auto-filled selection and have to confirm their choices before being able to continue. When participants have made their choice on this coarse list, we ‘zoom-in’ on the time interval around their switching point. Participants then see a 6-item choice list with steps *Δt* equal to one week. They make their fine-grained selection by choosing from the one-week steps in between the two options that marked their switching point on the coarse list. Figure 2 shows an example of a zoomed-in list that appeared after indicating a switching point after 10 weeks on the coarse list (*5-week)*. Note that choices in both iterations together result in a complete revelation of the preference for timed payments from the initial delay after the date of survey (5 or 8 weeks) to the longest payment delay after the date of survey (55 to 58 weeks).

Further note that in the coarse list, the first row was pre-set such that subjects were forced to choose the higher payment. In the fine list, the two end-point comparisons derived from the coarse list were also pre-set. While we allow participants to switch at most once, it is permissible to never switch. A zoomed-in weekly choice list is not shown if a participant chooses Option B in the last row of the coarse list, because the participant’s switching point lies outside the range of our choice lists. Auto-filling is done on both the coarse and the fine choice lists.

Each of the two choice lists provides us with a switching point from the higher-later to the lower-sooner payoff-timing pair expressed in weeks.^3^ We calculate implied annualized discount rates (continuous discounting) based on the payoff differences and switching points for each participant.^4^ To measure impatience, we calculate the discount rate that is implied by the choices on the 5-week list, which was always presented first. The discount rate is given by^5^:

$$\text{r}=\frac{52\bullet \text{ln}(1.15)}{{t}_{5}-5},$$

and a higher discount rate is associated with greater impatience (t_5_ is the switching point on the 5-week list).

Time inconsistent behavior is hypothesized to be driven by changes in the level of impatience (Rohde, 2019). We use the two switching points elicited using the 5-week (t_5_) and the 8-week (t_8_) choice lists to calculate the Decreasing Impatience Index (DI-index; Rohde, 2019) as:

$$DI=\frac{\left({t}_{8}-{t}_{5}\right)-3}{3\left({t}_{5}-5\right)},$$

with a first-list delay of 5 weeks and additional delay of 3 weeks for the second list. In contrast to constant hyperbolic factors (e.g., Rohde, 2010), the DI-index allows to identify and measure the changes of the level of impatience independently of the level of impatience itself. While an index value of zero indicates constant impatience, lower values (DI < 0) indicate increasing impatience and higher values (DI > 0) indicate decreasing impatience.

As noted by Rohde (2019), the elicitation method may lead to very patient subjects always choosing Option B. This also happened in our study, and we scored these subjects as t_5_ = 55.5, respectively t_8_ = 58.5, and DI = 0 (if they were very patient in both lists). We decided to include the subjects in the regression analyses to not skew results by excluding the most patient people in the population sample. However, as we cannot be sure that these participants exhibit constant discounting despite their extensive patience, we include an indicator variable for these subjects in all regressions. This indicator captures any effects that are specific to this special group of subjects or their scoring. In univariate and descriptive analyses of the DI-index that cannot control for the imputation DI = 0 we do not include these data. For completeness, we include the analyses without excluding any participants and regressions without the additional indicator variable in Online Appendix C.

In addition, participants may indirectly violate the impatience assumption underlying both choice lists. This is the case if a participant switches from Option B (€115) to Option A (€100) at an absolutely later time (after more weeks of total waiting) in the first choice list with the 5-week delay than in the second choice list with the 8-week delay. We cannot calculate the DI-index for these participants (cf. Rohde, 2019).

### Questionnaires

We use the 8-item Short Grit Scale (Grit-S, Duckworth & Quinn, 2009) to measure all participants’ perseverance and orientation towards long-term goals. The scale has two primary factors, consistency of interests and perseverance of effort, and improves upon the psychometric properties of the earlier and longer Grit-O scale (Duckworth et al., 2007). The perseverance component has also been demonstrated to correlate significantly with a behavioral measure based on a real effort task (Gerhards & Gravert, 2021). The scale and its sub-scales are typically scored by taking the average of the responses to the Likert items, taking reversed items into account. For comparability to the existing literature, we calculate this score and refer to it as *DQ-Grit Score.* However, the assumption that the metric for Likert scales is comparable across questions is problematic. We therefore construct indices as alternative measures for our analysis. The main *Grit Score* comprises all items, while *Grit CoI* (items G1, G3, G5, G6) and *Grit PoE* (items G2, G4, G7, G8) only include the respective items. For every questionnaire item, we first conduct a median split (cf. Dohmen et al., 2023, footnote 5). We then build the scores by counting the number of above median responses for the respective items for each participant. For the overall *Grit Score*, the score ranges from 0 to 8, for *Grit CoI* and *Grit PoE* the scores range from 0 to 4.

In addition, we assess participants’ dissatisfaction with their behavior in regard to financial planning and spending decisions. Specifically, we ask them to indicate how strongly they agree or disagree (7-point Likert scales) with statements regarding their (long-term) financial planning, savings behavior, spending behavior, and overall satisfaction with their financial habits. From their responses, we construct a *Finance Dissatisfaction Score* by counting the above-median responses to the respective four items. Similarly, we measure participants’ dissatisfaction with their health-related behaviors by asking them to indicate how strongly they agree or disagree (7-point Likert scales) with statements concerning their (long term) health planning, physical activity, eating behavior, and overall satisfaction with their health-related habits. Again, the number of participants’ above-median responses to these four items is counted and constitute the *Health Dissatisfaction Score.*

One half of the participants first answers the questionnaire on finances, then answers the questionnaire on health behavior second. The other half of the participants encounters the two questionnaires in reversed order. The order of the questionnaires is randomly determined on the individual level. All questionnaires are reproduced in Online Appendix A.

### Procedures

The experiment and surveys were run on the LISS Panel,^6^ which consists of 5000 households and about 7500 individuals. The sample is representative of the Dutch population aged 16 and above. Panel members are invited, provided with internet access and a computer (if not available), and complete the questionnaires online. Questionnaires are administered monthly and take about 60 minutes to complete. The questionnaires consist of the LISS Core Study and regularly changing Assembled Studies. These are administered back-to-back. Respondents are paid €15 per hour of their time in addition to any payments resulting from tasks in the Assembled Studies. A total of 3421 individuals participated in our experiment of which 300 participants (about 9%) were randomly selected to be paid for the experiments. This random selection was implemented to allow for significant payments for the selected participants in the context of the long time horizons studied in our experiment.^7^ If a participant was selected for payment, one of the two experimental parts of the study (time preference or ambiguity attitude elicitation) was selected at random to be payoff relevant. If the time preference task was selected, we selected one of the two choice lists, *5-week* or *8-week*, and one of the weekly steps at random. The participant received a payment in accordance with their choice of the timed payoff in the selected decision. All payments were made as bank transfers by the LISS panel administration. As participants interact with LISS and receive payments regularly, there should be no trust issues regarding the delayed payoffs.

**Table 1:** Summary statistics

	Impatience	DI-index	DQ-Grit Score	Grit Score	Grit PoE	Grit CoE
Median	0.632	0	4.875	2	1	0
Mean	2.718	0.648	4.895	2.423	1.518	0.905
Std. dev	4.762	3.822	0.841	2.163	1.378	1.204
N	3421	2229	3370	3370	3370	3370

Note: Impatience = annual discount rate as implied by first choice list with a 5-week up-front delay; DQ-Grit Score $\in$ [1, 7]; Grit Score $\in \{0, \dots , 8\}$; Grit PoE and CoI $\in \{0, \dots , 4\}$. Extremely patient participants with imputed DI-index = 0 are excluded in the DI-index column.

**Table 2:** Deviations from constant discounting

	Rohde 1	Rohde 2	Our data
Up-front delay	0 weeks	2 weeks	5 weeks
Decreasing impatience (DI > 0)	0.457	0.396	0.334
Constant impatience (DI = 0)	0.298	0.231	0.431
Increasing impatience (DI < 0)	0.245	0.374	0.235
*N*	94	91	2229

Note: Shares of all participants reported; Rohde 1 and Rohde 2 are based on the two choice lists of the second experiment reported in Rohde (2019). We exclude extremely patient participants with imputed DI-index = 0 from our data.

**Table 3:** Demographic correlates of time preference and grit

	(1)	(2)	(3)	(4)	(5)	(6)
	Impatience	Increasing Impatience	Decreasing Impatience	Grit Score	Grit CoI	Grit PoE
Female	− 0.163 (0.186)	0.002 (0.002)	0.354* (0.163)	0.189* (0.088)	0.087 (0.056)	0.103* (0.049)
Age	0.085* (0.034)	− 0.000 (0.000)	− 0.017 (0.029)	0.026 (0.016)	0.017 (0.010)	0.009 (0.009)
Age squared	− 0.000 (0.000)	− 0.000 (0.000)	0.000 (0.000)	− 0.000 (0.000)	− 0.000 (0.000)	− 0.000 (0.000)
Married	− 0.046 (0.216)	− 0.001 (0.002)	− 0.374* (0.190)	0.204* (0.102)	0.098 (0.065)	0.106 (0.057)
Divorced	0.318 (0.318)	− 0.005 (0.003)	− 0.591* (0.279)	0.125 (0.150)	0.021 (0.095)	0.103 (0.084)
High education	− 0.660*** (0.195)	− 0.001 (0.002)	− 0.167 (0.171)	0.210* (0.092)	0.221*** (0.058)	− 0.011 (0.051)
No. of children	0.171 (0.096)	− 0.000 (0.001)	0.135 (0.084)	− 0.005 (0.045)	− 0.011 (0.029)	0.006 (0.025)
Home ownership	− 0.717*** (0.206)	− 0.001 (0.002)	− 0.230 (0.180)	− 0.022 (0.096)	0.068 (0.061)	− 0.090 (0.054)
Log net income	− 0.527*** (0.157)	0.001 (0.001)	0.171 (0.134)	0.217** (0.074)	0.067 (0.047)	0.150*** (0.041)
Self-employed	0.210 (0.398)	0.002 (0.004)	0.649 (0.349)	0.471* (0.188)	0.072 (0.119)	0.399*** (0.105)
Adjusted *R*^2^	0.034	0.032	0.013	0.020	0.025	0.012
Observations	2903	2346	2346	2855	2855	2855

Note: OLS with standard errors in parentheses; Increasing Impatience = absolute value of DI-index if negative; Decreasing Impatience = DI-index if positive; CoI = Consistency of Interests Grit subscale, PoE = Perseverance of Effort Grit subscale; High education is an indicator for above median education. Models 2 and 3 do not include patient respondents with imputed DI = 0, which trivially implies consistency. */**/*** denote significance of difference from zero at 5% / 1% / 0.1%.

**Table 4:** Rank correlation coefficients

	Impatience	Increasing Impatience	Decreasing Impatience	Grit Score	Grit CoI
Inc. Impatience	− 0.26***				
Dec. Impatience	0.00	− 0.37***			
Grit Score	− 0.05*	0.04	− 0.01		
Grit CoI	− 0.04*	0.05	− 0.03	0.88***	
Grit PoE	− 0.04*	0.01	0.01	0.75***	0.39***

Note: Spearman’s rank correlation coefficients; Impatience = discount rate as implied by first choice list with 5-week up-front delay; CoI = Consistency of Interests Grit subscale; PoE = Perseverance of Effort Grit subscale; extremely patient participants with imputed DI-index = 0 are excluded; */**/*** denote significance of difference from zero at 5% / 1% / 0.1%.

**Table 5:** Explaining financial dissatisfaction score

	(1)	(2)	(3)	(4)	(5)
Impatience Z	0.055* (0.027)	0.034 (0.030)	0.053* (0.026)	0.034 (0.029)	0.034 (0.029)
Inc. impatience Z	0.047 (0.027)	0.016 (0.031)	0.044 (0.026)	0.016 (0.030)	0.016 (0.030)
Dec. impatience Z	− 0.037 (0.041)	− 0.029 (0.043)	− 0.040 (0.040)	− 0.029 (0.042)	− 0.035 (0.042)
Grit Score			− 0.150*** (0.012)	− 0.108*** (0.013)	
Grit PoE					− 0.000 (0.026)
Grit CoI					− 0.199*** (0.023)
Female		− 0.016 (0.065)		− 0.007 (0.064)	− 0.016 (0.064)
Age		0.004 (0.012)		0.009 (0.012)	0.011 (0.012)
Age squared		− 0.000* (0.000)		− 0.000* (0.000)	− 0.000* (0.000)
Married		− 0.090 (0.073)		− 0.065 (0.072)	− 0.063 (0.072)
Divorced		− 0.018 (0.105)		0.005 (0.103)	0.003 (0.103)
High education		− 0.121 (0.067)		− 0.104 (0.065)	− 0.082 (0.065)
No. of children		0.057 (0.034)		0.052 (0.034)	0.049 (0.033)
Home ownership		− 0.166* (0.071)		− 0.182** (0.070)	− 0.173* (0.070)
Log net income		0.006 (0.059)		0.025 (0.058)	0.014 (0.057)
Self-employed		0.023 (0.135)		0.074 (0.133)	0.036 (0.133)
Ladder of life		− 0.094*** (0.025)		− 0.074** (0.025)	− 0.067** (0.025)
High Ease of Living		− 0.560*** (0.066)		− 0.535*** (0.065)	− 0.529*** (0.064)
*Adj. R*^*2*^	0.017	0.158	0.070	0.186	0.195
*N*	2713	1982	2712	1981	1981

Note: OLS; standard errors in parentheses; dependent variable is the financial dissatisfaction index; impatience measures are z-scores; CoI = Consistency of Interests Grit subscale, PoE = Perseverance of Effort Grit subscale; education and ease of living variables are indicators for above median scores; all models include a dummy for DI = 0 for patient respondents waiting until the maximum delay of the choice list (see Section 2); reduced sample sizes due to missing variables for some participants; */**/*** denote significance of difference from zero at 5% / 1% / 0.1%.

**Table 6:** Explaining health dissatisfaction score

	(1)	(2)	(3)	(4)	(5)
Impatience Z	− 0.018 (0.027)	− 0.014 (0.037)	− 0.019 (0.027)	− 0.015 (0.037)	− 0.016 (0.037)
Inc. impatience Z	0.021 (0.027)	− 0.013 (0.041)	0.018 (0.027)	− 0.014 (0.041)	− 0.013 (0.041)
Dec. impatience Z	− 0.017 (0.041)	0.029 (0.051)	− 0.019 (0.041)	0.022 (0.051)	0.014 (0.051)
Grit Score			− 0.086*** (0.012)	− 0.056*** (0.016)	
Grit PoE					0.036 (0.032)
Grit CoI					− 0.130*** (0.027)
Female		− 0.183* (0.077)		− 0.178* (0.076)	− 0.175* (0.076)
Age		0.007 (0.014)		0.010 (0.014)	0.010 (0.014)
Age squared		− 0.000 (0.000)		− 0.000 (0.000)	− 0.000 (0.000)
Married		− 0.031 (0.089)		− 0.017 (0.089)	− 0.017 (0.089)
Divorced		0.146 (0.128)		0.155 (0.127)	0.153 (0.127)
High education		− 0.045 (0.078)		− 0.033 (0.078)	− 0.017 (0.078)
No. of children		− 0.023 (0.041)		− 0.024 (0.040)	− 0.027 (0.040)
Home ownership		− 0.078 (0.087)		− 0.083 (0.087)	− 0.064 (0.087)
Log net income		0.200** (0.064)		0.210*** (0.064)	0.205** (0.064)
Self-employed		0.021 (0.156)		0.046 (0.155)	0.017 (0.155)
High general health		− 0.648*** (0.081)		− 0.601*** (0.082)	− 0.611*** (0.082)
Sick days		0.112** (0.042)		0.107** (0.042)	0.100* (0.041)
Smoking		0.301** (0.105)		0.333** (0.105)	0.331** (0.104)
Alcohol days		− 0.014 (0.018)		− 0.015 (0.017)	− 0.013 (0.017)
Activity days		− 0.109*** (0.021)		− 0.109*** (0.021)	− 0.110*** (0.021)
Walking days		− 0.050*** (0.014)		− 0.049*** (0.014)	− 0.051*** (0.014)
*Adj. R*^*2*^	− 0.001	0.116	0.017	0.123	0.130
*N*	2712	1397	2712	1397	1397

Note: OLS; standard errors in parentheses; dependent variable is the health dissatisfaction index; impatience measures are z-scores; CoI = Consistency of Interests Grit subscale, PoE = Perseverance of Effort Grit subscale; education and general health are indicators for above median scores; all models include a dummy for DI = 0 for patient respondents waiting until the maximum delay of the choice list (see Section 2); reduced sample sizes due to missing variables for some participants; */**/*** denote significance of difference from zero at 5% / 1% / 0.1%.

**Table 7:** Explaining financial outcomes

	(1)	(2)	(3)	(4)	(5)	(6)	(7)
	Balance on Bank Accounts	Sum of Investments	Log Net Inc	High Fin. Satis	Ladder	High Ease of Living	Home Ownership
Impatience Z	− 7.733** (2.492)	− 1.545 (12.846)	− 0.070*** (0.013)	− 0.031** (0.010)	− 0.088*** (0.026)	− 0.041*** (0.010)	− 0.026** (0.009)
Inc. impatience Z	− 1.339 (2.483)	3.849 (16.617)	− 0.000 (0.013)	− 0.011 (0.010)	− 0.037 (0.026)	− 0.019 (0.010)	− 0.009 (0.009)
Dec. impatience Z	− 1.162 (3.042)	− 28.613 (34.621)	0.023 (0.019)	− 0.026 (0.015)	0.010 (0.040)	− 0.026 (0.015)	− 0.009 (0.013)
Grit PoE	− 1.921 (1.834)	9.265 (7.265)	0.028* (0.011)	0.007 (0.009)	− 0.010 (0.023)	0.004 (0.009)	− 0.018* (0.008)
Grit CoI	− 0.302 (1.604)	0.157 (6.131)	0.016 (0.010)	0.048*** (0.008)	0.136*** (0.020)	0.037*** (0.008)	0.021** (0.007)
Female	− 6.881 (4.205)	− 13.045 (17.103)	− 0.458*** (0.026)	− 0.002 (0.019)	0.012 (0.052)	− 0.055** (0.021)	− 0.037* (0.017)
Age	1.958** (0.668)	5.338* (2.673)	0.061*** (0.004)	0.003 (0.003)	0.004 (0.008)	0.001 (0.004)	0.013*** (0.003)
Age squared	− 0.011 (0.007)	− 0.041 (0.025)	− 0.001*** (0.000)	0.000 (0.000)	0.000 (0.000)	− 0.000 (0.000)	− 0.000*** (0.000)
*Adj. R*^*2*^	0.052	0.025	0.212	0.056	0.038	0.042	0.021
*N*	1138	254	2313	2399	2480	2244	2708

Note: OLS; standard errors in parentheses; dependent variables Balance on Bank Accounts and Sum of Investments in thousand euros; impatience measures are z-scores; CoI = Consistency of Interests Grit subscale, PoE = Perseverance of Effort Grit subscale; all models include a dummy for DI = 0 for patient respondents waiting until the maximum delay of the choice list (see Section 2); reduced sample sizes due to missing data for some dependent variables; */**/*** denote significance of difference from zero at 5% / 1% / 0.1%.

### Demographic controls and LISS modules for finance and health

We augment our experimental data with selected variables from the LISS Core Study and its modules ‘Health’,^8^ ‘Assets’,^9^ and ‘Income’.^10^ These are used as control variables (education and net income) and as additional dependent variables. (When we control for the level of education, we include the education categories of Statistics Netherlands (1 = primary school to 6 = university). To control for income, we use the logarithm of participant’s net income.^11^

**Table 8:** Explaining health outcomes

	(1)	(2)	(3)	(4)	(5)	(6)
	High General Health	Sick days per month	Smoking	Alcohol intake, days per week	Physically active, days per week	Walking > 10 m, days per week
Impatience Z	− 0.020* (0.008)	0.013 (0.019)	0.014* (0.007)	0.012 (0.053)	− 0.008 (0.032)	− 0.104* (0.050)
Inc. impatience Z	− 0.006 (0.008)	− 0.002 (0.019)	0.008 (0.007)	− 0.125* (0.059)	0.022 (0.033)	0.019 (0.051)
Dec. impatience Z	0.010 (0.012)	0.000 (0.029)	− 0.011 (0.010)	− 0.115 (0.074)	0.022 (0.050)	− 0.101 (0.077)
Grit PoE	0.035*** (0.007)	− 0.007 (0.017)	0.007 (0.006)	− 0.055 (0.046)	0.043 (0.029)	0.140** (0.045)
Grit CoI	0.031*** (0.006)	− 0.043** (0.015)	0.002 (0.005)	0.052 (0.039)	− 0.011 (0.026)	0.010 (0.040)
Female	0.010 (0.022)	− 0.053 (0.051)	− 0.035* (0.018)	0.042 (0.129)	0.076 (0.088)	0.090 (0.136)
Age	− 0.050** (0.016)	0.134*** (0.038)	− 0.029* (0.013)	− 0.503*** (0.100)	− 0.399*** (0.065)	0.266** (0.100)
Age squared	− 0.016*** (0.002)	0.003 (0.006)	0.005** (0.002)	0.004 (0.016)	− 0.006 (0.010)	0.013 (0.015)
*Adj. R*^*2*^	0.095	0.010	0.008	0.134	0.045	0.008
*N*	2680	2678	2678	1583	2677	2677

Note: OLS; standard errors in parentheses; High General Health is an indicator for an above median general health question response; impatience variables are z-scores; CoI = Consistency of Interests Grit subscale, PoE = Perseverance of Effort Grit subscale; all models include a dummy for DI = 0 for patient respondents waiting until the maximum delay of the choice list (see Section 2); reduced sample sizes due to missing data for some dependent variables; */**/*** denote significance of difference from zero at 5% / 1% / 0.1%.

There are seven finance outcome variables, of which three measure participants’ assets: The sum on bank accounts represents the total balance of checking, savings, and term deposit accounts as well as savings bonds, savings certificates, and savings schemes at the end of 2017. The sum of investments covers growth funds, share funds, bonds, debentures, stocks, options and warrants at the end of the same year. Both variables can be positive or negative. We also include an indicator variable for owning their home. In addition, there are two measures related to income: Net income as defined before and an 11-point Likert type question asking the participants to indicate how hard or easy it is for them to live off their income (0 = very hard to 10 = very easy). We conduct a median split for the ease of living variable and include an indicator variable for above median responses in our analyses. Finally, participants are asked how satisfied they are with their financial situation (0 = not at all satisfied to 10 = entirely satisfied). Again, we conduct the median split and binarize the response. Finally, we ask where participants would place themselves on an imaginary ‘ladder of life’. The bottom of the ladder represents the worst possible life while the top represents the best possible life (0 to 10, higher is better). These questions, while less specific, are conceptually quite similar to our dedicated financial dissatisfaction scale.

We also include six additional health outcome variables. The first targets general health and uses a 5-point Likert scale. Participants indicate how they would describe their health in general (1 = poor, 2 = moderate, 3 = good, 4 = very good, 5 = excellent). We conduct a median split for inclusion in our analyses. In addition, we use the self-reported number of days of the last month on which participants were unable to work, go to school, or do housekeeping work because of illness (1 = 0 days, 2 = 1 or 2 days, 3 = 3 to 5 days, 4 = 5 to 10 days, 5 = more than 10 days). Next, we include an indicator for smoking and a question about the number of days in the last week that alcohol was consumed (0 to 7), which both aim to capture current health-related decision making. Finally, we include two questions on physical activity. We ask on how many of the past 7 days participants engaged in strenuous physical activity (lifting heavy loads, digging, aerobics, cycling, etc.; 0–7) and on how many days they spent at least 10 minutes walking (0–7).

## Results: Time preference and grit

We first provide descriptive statistics, study variation in demographics, and then the correlation of the time preference with the grit measures. Table 1 shows the summary statistics of our key variables of interest: impatience, the DI-index, and the Grit measures. The median participant switches from Option B to Option A after 16 weeks, implying a discount rate of 0.63 and moderate impatience. In terms of the DI-index, the median is zero, with the mean being positive at 0.65. There appears to be a tendency to towards decreasing impatience. We observe a median DQ-Grit Score of 4.875 with mean 4.9, which is relatively high on the 7-point scale. For comparison, Duckworth and Quinn (2009) report a grand mean of 3.4 for a large online sample of participants aged 25 up to 65 +. Internal reliability is high with Cronbach’s alpha approaching 0.78 for the 8-item overall score (0.75 for the 4-item CoI, 0.77 for the 4-item PoE). For the Grit Score, the median is 2 and the mean is 2.423 (scale 0 to 8). The median and mean of Grit PoE are 1 and 1.518 (scale 0 to 4), respectively. Both are higher than the corresponding values of Grit CoI, which are 0 and 0.905 (scale 0 to 4), respectively.^12^

Table 2 takes a closer look at the DI-index. The table presents the share of participants that exhibit decreasing, constant, or increasing impatience. For comparability, we show our data along-side values that have been reported in Rohde (2019). Constant impatience is relatively prevalent in our sample, with about 43% of participants not showing signs of increasing or decreasing impatience across our two choice lists. A significantly larger share of participants exhibits decreasing impatience than increasing impatience (33.4% vs. 23.5%, p < 0.0001, sign test). Interestingly, there seems to be a decreasing relationship between the number of weeks of up-front delay and the prevalence of decreasing impatience. Neglecting the differences in samples, Rohde finds 46% for 0 weeks of delay and 40% for 2 weeks of delay, while we find just 33% for 5 weeks of up-front delay. No systematic pattern stands out from constant and increasing impatience data.

To identify how demographic characteristics are associated with our measures of impatience, time inconsistency, and grit, we run individual regressions of these variables on a basic set of individual- and household-level background variables available on the LISS panel (see Section 2.4). Table 3 shows the results of these regressions. In terms of time preferences, we find impatience (model 1) to be significantly positively related to age. We find a significant negative association with being highly educated, home ownership, and net income, which is conceptually in line with previous studies (see literature overview in Meissner et al., 2023).

The DI-index extends both in the negative and the positive domain and expresses different deviations from constant discounting. As such, effects of the background variables on the DI-index are hard to interpret. To avoid this complication, we split the DI-index at zero into its positive and negative two components. While an index value of zero expresses time consistency, positive values of the index express degrees of decreasing impatience and negative values express degrees of increasing impatience. In the subsequent analysis we include two variables to capture the two components separately: Decreasing Impatience matches the DI-index when it is positive. Increasing impatience matches the absolute value of the DI-index when it is negative. Increasing impatience (Table 3, model 2) is not statistically significantly associated with any background variable. Decreasing impatience (model 3) is significantly positively related to female gender and negatively with being married or divorced.

Models 4—6 study the main Grit Score and its consistency of interest and perseverance of effort components. In our representative sample of the Dutch population, above median grit is significantly positively correlated with being female, being married, above median educational attainment, net income, and being self employed (model 4). Models 5 and 6 reveal that the correlation with high education appears only for the CoI component, while the gender, income and self-employment factors mainly associate with the Grit Score through the perseverance of effort component. The observed association with gender is not commonly observed (Credé et al., 2017).

Table 4 shows raw rank correlation coefficients between our key variables of interest. An above median Grit Score is significantly negatively correlated with impatience. That is, a strong long-term goal orientation is related to lower impatience. The relationship to impatience is driven by both the CoI and PoE components of grit. None of the grit measures correlates with the time inconsistency measures. That is, the hypothesized relationship between time inconsistency and the grit consistency of interests facet is not supported in the data. Table B1 in Online Appendix B shows the correlations for each individual Grit-S item.

## Results: Predictive power for financial and health dissatisfaction and outcomes

In a next step, we study whether and how the time preference and grit measures are useful in predicting panel participants’ financial and health outcomes, and their subjective assessment of these outcomes. Impatience, time inconsistency, as well as low cognitive control (as potentially captured by low grit) have been related in various ways theoretically and empirically with poor financial and health outcomes (see discussion in Sutter et al., 2013). Importantly, it is typically assumed that people aim for better outcomes but fail to achieve them due to intertemporally suboptimal behavior. Thus, the items of our dissatisfaction scores are designed to identify such failure to realize financial and health goals due to poor intertemporal decision making. We first look at the survey measures for dissatisfaction and then consider a broader set of variables available through the LISS panel modules on health and financial assets.

Both dissatisfaction scores have relatively high internal reliability scores with Cronbach’s alpha reaching 0.78 and 0.8 for the 4-item Finance and Health questionnaires, respectively. We observe substantial variation in both Financial Dissatisfaction (median = 2, mean = 1.73, sd = 1.40) and Health Dissatisfaction (median = 1, mean = 1.42, sd = 1.37), and the Financial Dissatisfaction Score is significantly higher than Health Dissatisfaction Score (z = 10.61, p < 0.001, Wilcoxon signed rank test).

Table 5 shows results for the Financial Dissatisfaction Score. In models 1 and 2, we regress financial dissatisfaction on impatience and the time inconsistency measures, with and without additional controls to capture their demographics and life circumstances. To aid the interpretation of coefficient magnitudes, we z-standardize the time preference measures when including them as explanatory variables. Without controls, we observe a positive correlation between impatience and financial dissatisfaction. This correlation vanishes when controls are added. Models 3 and 4 add the Grit Score to the analysis. Higher values of Grit are associated with significantly lower dissatisfaction. When splitting grit into its two components in model 5, we observe that the additional explanatory power of grit for financial dissatisfaction stems from consistency of interests, rather than the perseverance of effort.

Table 6 shows results for the Health Dissatisfaction Score. We observe a similar pattern of associations, with grit being strongly negatively associated with health dissatisfaction. Splitting grit into its two components (model 5) reveals that especially the CoI component has explanatory power for health dissatisfaction. Impatience and the time inconsistency measures do not have predictive power for health dissatisfaction in any of the specifications. Notably, our controls include measure of actual health outcomes such as a measure of above median general health and the number of sick days. Even after controlling for these actual life outcomes Grit still has merit in explaining health dissatisfaction. In sum, for both dissatisfaction scores, even after controlling for demographic characteristics, grit—and especially its CoI component—has significant explanatory power for participants’ dissatisfaction with their financial and health-related behavior.

We next consider the relationship between impatience, time inconsistency, and grit with several measures of participants’ financial and health outcomes available on the LISS panel. We report results for the disaggregated Grit PoE and CoI scores. Results using the overall Grit Score are available in Online Appendix B, Tables B2 and B4. Table 7 shows results for financial outcomes. Impatient participants hold lower bank account balances, have lower income, are less satisfied with their overall financial situation, place themselves lower in social status, find it more difficult to make ends meet, and are less likely to own their home. There are no associations with increasing or decreasing impatience. Both components of Grit are associated with financial outcomes. Perseverance of effort relates positively to net income, but negatively to home ownership. Consistency of interests relates positively to financial satisfaction, self-perceived social status, making ends meet, and home ownership.

Table 8 shows results for health outcomes. Impatience is negatively associated with general health and the number of days walking more than 10 minutes. It is positively correlated to smoking. We only observe a single negative association of the two time inconsistency measures with alcohol consumption. Perseverance of effort relates positively to general health and the frequency of taking walks. Consistency of interests relates positively to general health and fewer sick days.

## Discussion & conclusion

We present the first joint measurement of time preferences, including time inconsistency, and grit on a large representative sample, and combine these measures with a survey of participants’ dissatisfaction with their financial and health related behaviors, as well as a broader set of financial and health outcomes. Participants are on average impatient and about 56% deviate from constant discounting, and more often in the direction of decreasing impatience. Impatience is predictive of several financial outcomes, and to a smaller degree of health outcomes. In contrast, we do not observe any associations for increasing or decreasing impatience with dissatisfaction measures or broader outcomes available on the LISS panel for health and finance. Grit has strong predictive power for financial and health-related decision making that goes beyond impatience alone. The higher participants’ score in grit, the less dissatisfied they are with their behaviors, and especially the CoI component of grit predicts several financial and health outcomes, after controlling for impatience and demographic characteristics.

Correlations of grit with the demographic variables of our sample are stronger than in earlier research. Specifically, we find female gender, being married, high educational attainment, and net income to be significantly positively correlated with grit. However, the effect of high education manifests itself through both components of grit, while gender, marital status, and income mainly determine perseverance of effort. The results are in line with the meta-analysis of Credé et al., 2017 who also report small correlations for these variables.

Two important insights emerge regarding the relationship between the psychological grit scale and the incentivized economic time preference measures. First, as predicted, impatience correlates with the perseverance of effort component of Grit-S. However, the hypothesized relationship between the consistency of interests component of grit and deviations from constant discounting does not materialize in our data. A possible explanation for this lies in the framework we use to measure time preferences and time inconsistency. As most of the empirical literature in economics, we utilize the Money Earlier or Later (MEL) framework (Cohen et al., 2020). Its defining characteristic is that decisions are made over differently timed *cash flow events*, rather than *consumption events* per se. While impatience estimated in the MEL framework generally predicts field behavior in various contexts, the evidence for effects of time inconsistent choices (such as present bias) is less compelling (see Cohen et al., 2020 for a review). For example, Augenblick et al. (2015) find inconsistency to be limited in choices over monetary rewards, but substantially stronger in real effort task behavior. It has even been argued that choices in the MEL framework might be better explained by heuristics than discounting models (Marzilli Ericson et al., 2015). Our results closely match these previous observations: Impatience shows predictive power, but time inconsistency fails to add much value. The latter aspect is consistent with the low correlation of time inconsistency with the (highly predictive) CoI component of grit.

Second, despite the correlation of impatience and grit, in particular its PoE component, we find that both are predictive of participants’ dissatisfaction with their financial behavior and their financial outcomes more broadly. That is, both measures seem to tap into different traits, both relevant to intertemporal financial decision making and consistency in planning (cf. Borghans et al., 2008). However, the economic time preference measures perform poorly for health behaviors and outcomes, while grit is predictive in both the health and the financial domain. Comparing the two components of grit, we find that the consistency of interests component is more predictive than the perseverance of effort component for the two dissatisfaction measures; for broader measures of financial and health outcomes both components tend to be equally predictive. The strong performance for our dissatisfaction measures suggests that, as intended, these items tap into dissatisfaction deriving from an inconsistency between planned and actual behavior in the two domains of health and finances. Moffitt et al. (2011) show that childhood self-control is a strong predictor of life outcomes across a broad range of outcomes, including health. Our results suggest that the discounting measures of time preference as typically employed in economics do not fully capture such psychological self-control effects.

In conclusion, the simple-to-administer grit measure seems to comprise additional psychological aspects that are not well captured by the standard set of economic time preference measures. How strongly these aspects help to explain behavior, however, may depend on the specific context. Our results suggest that empirical studies on intertemporal economic and financial decisions may strongly benefit from including the Grit-S Scale over and above any traditional time preference measures based on monetary time trade-offs.

## Supplementary Information

Below is the link to the electronic supplementary material.

Supplementary file1 (DOCX 63 KB)

## Data Availability

Analysis files and our experimental data are available on OSF: https://osf.io/qd63b/. This paper uses LISS panel data. More information about the panel can be found at https://www.lissdata.nl.

## Footnotes

1 It has also been argued that deviations from constant discounting may be a symptom of non-linear time perception (Bradford et al., 2019). In addition, there have been attempts to tailor the elicitation of time preferences to the domain under study, for example by directly measuring time preferences over health events (see Bleichrodt et al., 2016 for an overview) or environmental policy choices (Viscusi et al., 2008).

2 There is also some debate about the factor structure of the Grit scales, both in the long and short forms. The interested reader may refer to Credé et al. (2017), Guo et al. (2019), Jachimowicz et al. (2018, 2019), Disabato et al. (2019), and Duckworth et al. (2021).

3 Technically, we add 0.5 to all switching points expressed in weeks. This is to account for the indifference lying between the week the switch occurs after and the subsequent week. We use the midpoint between the two weeks as an approximation.

4 We assume that over the observed time range consumption is approximately constant, and that experimental payments are low relative to total income, implying locally linear utility.

5 Implied by the indifferenc $\EUR100\cdot e^{-r\cdot\frac5{52}}=\EUR115\cdot e^{-r\cdot\frac{t_5}{52}}$ if the switching row is given by *t*_*5*_.

6 LISS stands for Longitudinal Internet studies for the Social Sciences. The panel is organized and maintained by CentERdata in the Netherlands. More information, as well as the extensive data archive, is available on their website: https://www.lissdata.nl.

7 March et al. (2016) show that high nominal payoffs are more important for the effects of monetary incentives than a high selection probability. Our payoffs are comparatively large and meaningful for the general population sample. Noussair et al. (2014) report on an experiment conducted on the LISS panel and in the laboratory. Like us, they pay a random sample of panel participants but also include a sample of laboratory participants in which everyone was paid. Their results do not differ substantially between the two samples.

8 Wave 11, items ch18k004, ch18k099, ch18k125, ch18k126, ch18k135, ch18k185, ch18k191.

9 Wave 6, items ca18f012, ca18f016.

10 Wave 11, items ci18k006, cu18k005, ci18k244.

11 Variable: nettoink, describing monthly net (post-tax) income in Euros. If not provided by the participant, this value is imputed from net income categories (nettocat) by using the average of the indicated category.

12 The time preference choice lists were the first part of the three-part study. We observe very minor attrition between parts. While 3421 participants filled-in the first choice list, 3414 (7 less) participants also completed the second choice list. In part two, we lost another 44 participants and ended up with 3370 completed Grit-S questionnaires.
