# Supplementary material for: Grit, discounting, & time inconsistency
Source: J Risk Uncertain. 2025 Jun 17;70(3):201–23. doi: 10.1007/s11166-025-09456-8 (PMC12198323; doi:10.1007/s11166-025-09456-8)
Supplement: Supplementary file 1 — Supplementary file1 (DOCX 63 KB) [file 11166_2025_9456_MOESM1_ESM.docx]

# Online Appendix

## Section A: Questionnaires

### Grit-S

These are a number of statements about how you tackle difficult tasks and projects compared to other people you know. There are no right or wrong answers: We would like to know how you see yourself.

G1: New ideas and projects sometimes distract me from previous ones.

very much like me O O O O O O O not like me at all

G2: Setbacks don’t discourage me. I don’t give up easily.

very much like me O O O O O O O not like me at all

G3: I have been obsessed with a certain idea or project for a short time but later lost interest.

very much like me O O O O O O O not like me at all

G4: I am a hard worker.

very much like me O O O O O O O not like me at all

G5: I often set a goal but later choose to pursue a different one.

very much like me O O O O O O O not like me at all

G6: I have difficulty maintaining focus on projects that take more than a few months to complete.

very much like me O O O O O O O not like me at all

G7: I finish whatever I begin.

very much like me O O O O O O O not like me at all

G8: I am diligent.

very much like me O O O O O O O not like me at all

*Note: This questionnaire is taken from Duckworth and Quinn (2009). Items G1, G3, G5, G6 enter the score in reverse and constitute the consistency-of-interests factor of Grit, while the remaining items target perseverance of effort.*

### Financial Dissatisfaction Questionnaire

We would like to give you a brief overview of your views on finances.

F1: I think I should spend more time on financial planning, especially with regard to my pension.

strongly agree O O O O O O O strongly disagree

F2: I think I should save more (eg through a voluntary extra pension) in order to have sufficient income in the future.

strongly agree O O O O O O O strongly disagree

F3: I often think that I should spend money more thoughtfully in my daily consumption.

strongly agree O O O O O O O strongly disagree

F4: I am completely satisfied with my financial planning and my daily spending habits.

strongly agree O O O O O O O strongly disagree

## *Note: Item F4 enters the Financial Dissatisfaction Score in reverse.*

### Health Dissatisfaction Questionnaire

We would like to give you a brief overview of your views on health topics.

H1: I think I should spend more time thinking about my lifestyle and health.

strongly agree O O O O O O O strongly disagree

H2: I think I should spend more time on sports and other physical activities.

strongly agree O O O O O O O strongly disagree

H3: I often think I should eat more thoughtfully considering my health.

strongly agree O O O O O O O strongly disagree

H4: I am completely satisfied with my lifestyle (eating patterns and physical activities).

strongly agree O O O O O O O strongly disagree

## *Note: Item H4 enters the Health Dissatisfaction Score in reverse.*

## Section B: Additional Results

Table B1: Correlations of Grit-S items and impatience measures

|  | Impatience | DI Index | Increasing Impatience | Decreasing Impatience |
| --- | --- | --- | --- | --- |
| G1 CoI (new ideas) | -0.08*** | 0.01 | 0.02 | 0.03 |
| G3 CoI (lose interest) | 0.02 | 0.04 | -0.03 | 0.04 |
| G5 CoI (switch goals) | 0.05* | 0.03 | -0.01 | 0.03 |
| G6 CoI (focus) | 0.05* | 0.02 | -0.01 | 0.03 |
| G2 PoE (setbacks) | -0.05* | 0.01 | 0.00 | 0.01 |
| G4 PoE (hard worker) | -0.11*** | -0.07** | 0.09*** | -0.04 |
| G7 PoE (finish projects) | -0.06** | -0.05* | 0.05* | -0.03 |
| G8 PoE (diligent) | -0.08*** | -0.04 | 0.04* | -0.02 |

Notes: Spearman’s rank correlation coefficients; Impatience = discount rate as implied by first choice list with 5-week up-front delay; CoI = Consistency of Interests Grit subscale; PoE = Perseverance of Effort Grit subscale; extremely patient participants with imputed DI-index = 0 are excluded; */**/*** denote significance of difference from zero at 5% / 1% / 0.1%.

Table B2: Explaining financial outcomes (overall Grit Score)

|  | (1) | (2) | (3) | (4) | (5) | (6) | (7) |
| --- | --- | --- | --- | --- | --- | --- | --- |
|  | Balance on Bank Accounts | Sum of Investments | Log Net Income | High Fin. Satis. | Ladder of Life | High Ease of Living | Home Ownership |
| Impatience Z | -7.734** (2.491) | -1.990 (12.826) | -0.070*** (0.013) | -0.031** (0.010) | -0.088*** (0.026) | -0.041*** (0.010) | -0.026** (0.009) |
| Inc. impatience Z | -1.285 (2.480) | 3.508 (16.601) | -0.000 (0.013) | -0.011 (0.010) | -0.035 (0.026) | -0.019 (0.010) | -0.009 (0.009) |
| Dec. impatience Z | -1.159 (3.041) | -28.476 (34.598) | 0.023 (0.019) | -0.028 (0.015) | 0.004 (0.040) | -0.027 (0.015) | -0.011 (0.013) |
| Grit Score | -1.032 (0.972) | 4.146 (3.752) | 0.022*** (0.006) | 0.029*** (0.005) | 0.070*** (0.012) | 0.022*** (0.005) | 0.003 (0.004) |
| Female | -6.939 (4.202) | -12.937 (17.092) | -0.458*** (0.026) | -0.003 (0.019) | 0.009 (0.052) | -0.056** (0.021) | -0.038* (0.017) |
| Age | 1.963** (0.668) | 5.123 (2.658) | 0.061*** (0.004) | 0.003 (0.003) | 0.004 (0.008) | 0.001 (0.004) | 0.013*** (0.003) |
| Age^2^ | -0.011 (0.007) | -0.040 (0.025) | -0.001*** (0.000) | 0.000 (0.000) | 0.000 (0.000) | -0.000 (0.000) | -0.000*** (0.000) |
| *Adj. R^2^* | 0.053 | 0.026 | 0.212 | 0.053 | 0.032 | 0.040 | 0.017 |
| *N* | 1138 | 254 | 2313 | 2399 | 2480 | 2244 | 2708 |

Notes: OLS. Standard errors in parentheses; impatience measures are z-scores; all models include a dummy for DI=0 for patient respondents waiting until the maximum delay of the choice list (see Section 2); dependent variables Balance on Bank Accounts and Sum of Investments in thousand euros; reduced sample sizes due to missing data for some dependent variables; */**/*** denote significance of difference from zero at 5% / 1% / 0.1%.

Table B3: Explaining financial outcomes (additional controls)

|  | (1) | (2) | (3) | (4) | (5) | (6) | (7) |
| --- | --- | --- | --- | --- | --- | --- | --- |
|  | Balance on Bank Accounts | Sum of Investments | Log Net Inc. | High Fin. Satis. | Life Ladder | High Ease of Living | Home Ownership |
| Impatience Z | -6.881** (2.464) | -1.242 (12.686) | -0.045*** (0.012) | -0.024* (0.010) | -0.067** (0.025) | -0.033*** (0.010) | -0.022** (0.008) |
| Inc. impatience Z | -0.523 (2.458) | -0.097 (16.513) | 0.004 (0.012) | -0.011 (0.010) | -0.035 (0.026) | -0.018 (0.010) | -0.009 (0.008) |
| Dec. impatience Z | -1.920 (3.008) | -24.585 (34.013) | 0.027 (0.018) | -0.024 (0.015) | 0.014 (0.039) | -0.024 (0.015) | -0.009 (0.013) |
| Grit PoE | -2.074 (1.818) | 7.422 (7.180) | 0.030** (0.011) | 0.011 (0.009) | -0.004 (0.023) | 0.007 (0.009) | -0.017* (0.007) |
| Grit CoI | -0.792 (1.591) | -1.018 (6.091) | -0.001 (0.009) | 0.041*** (0.008) | 0.117*** (0.020) | 0.029*** (0.008) | 0.015* (0.007) |
| Female | -3.316 (4.197) | -9.903 (16.963) | -0.433*** (0.024) | 0.014 (0.019) | 0.060 (0.051) | -0.036 (0.020) | -0.018 (0.016) |
| Age | 1.618* (0.708) | 4.864 (2.848) | 0.065*** (0.004) | -0.002 (0.003) | -0.013 (0.009) | 0.002 (0.004) | 0.001 (0.003) |
| Age squared | -0.008 (0.007) | -0.037 (0.026) | -0.001*** (0.000) | 0.000 (0.000) | 0.000* (0.000) | -0.000 (0.000) | -0.000 (0.000) |
| Married | 11.589* (5.114) | -5.663 (18.441) | -0.069* (0.029) | 0.101*** (0.024) | 0.287*** (0.064) | 0.062* (0.025) | 0.219*** (0.021) |
| Divorced | -23.211** (7.637) | -20.249 (33.983) | 0.034 (0.043) | -0.027 (0.037) | -0.120 (0.097) | -0.051 (0.037) | -0.034 (0.032) |
| High education | 11.185** (4.172) | 23.689 (16.214) | 0.471*** (0.025) | 0.134*** (0.020) | 0.404*** (0.054) | 0.145*** (0.021) | 0.108*** (0.017) |
| No. of children | -2.333 (2.298) | -9.197 (9.588) | -0.062*** (0.013) | -0.023* (0.010) | 0.019 (0.028) | -0.030** (0.011) | 0.065*** (0.009) |
| Self-employed | 16.588 (10.135) | 101.333** (30.884) | -0.092 (0.055) | -0.062 (0.046) | -0.039 (0.122) | -0.004 (0.048) | 0.062 (0.039) |
| *Adj. R^2^* | 0.080 | 0.065 | 0.333 | 0.082 | 0.070 | 0.066 | 0.115 |
| *N* | 1136 | 253 | 2310 | 2394 | 2475 | 2239 | 2702 |

Notes: OLS; standard errors in parentheses; dependent variables Balance on Bank Accounts and Sum of Investments in thousand euros; impatience measures are z-scores; CoI = Consistency of Interests Grit subscale, PoE = Perseverance of Effort Grit subscale; education is an indicator for above median scores; all models include a dummy for DI=0 for patient respondents waiting until the maximum delay of the choice list (see section 2); reduced sample sizes due to missing data for some dependent variables; */**/*** denote significance of difference from zero at 5% / 1% / 0.1%.

Table B4: Explaining health outcomes (overall Grit Score)

|  | (1) | (2) | (3) | (4) | (5) | (6) |
| --- | --- | --- | --- | --- | --- | --- |
|  | High General Health | Sick days per month | Smoker | Alcohol intake, days per week | Physically active, days per week | Walking > 10m, days per week |
| Impatience Z | -0.020* (0.008) | 0.013 (0.019) | 0.014* (0.007) | 0.011 (0.053) | -0.008 (0.032) | -0.103* (0.050) |
| Inc. impatience Z | -0.006 (0.008) | -0.002 (0.019) | 0.008 (0.007) | -0.124* (0.059) | 0.022 (0.033) | 0.018 (0.051) |
| Dec. impatience Z | 0.011 (0.012) | 0.001 (0.029) | -0.011 (0.010) | -0.120 (0.074) | 0.024 (0.050) | -0.096 (0.077) |
| Grit Score | 0.033*** (0.004) | -0.027** (0.009) | 0.004 (0.003) | 0.004 (0.023) | 0.014 (0.015) | 0.069** (0.023) |
| Female | -0.050** (0.016) | 0.135*** (0.038) | -0.029* (0.013) | -0.502*** (0.100) | -0.397*** (0.065) | 0.269** (0.100) |
| Age | -0.016*** (0.002) | 0.003 (0.006) | 0.005** (0.002) | 0.004 (0.016) | -0.006 (0.010) | 0.014 (0.015) |
| Age^2^ | 0.000*** (0.000) | -0.000 (0.000) | -0.000** (0.000) | 0.000* (0.000) | -0.000 (0.000) | -0.000 (0.000) |
| *Adj. R^2^* | 0.095 | 0.010 | 0.008 | 0.133 | 0.045 | 0.007 |
| *N* | 2680 | 2678 | 2678 | 1583 | 2677 | 2677 |

Notes: OLS; standard errors in parentheses; impatience measures are z-scores; High General Health is an indicator for an above median response; all models include a dummy for DI=0 for patient respondents waiting until the maximum delay of the choice list (see Section 2); reduced sample sizes due to missing data for some dependent variables; */**/*** denote significance of difference from zero at 5% / 1% / 0.1%

Table B5: Explaining health outcomes (additional controls)

|  | (1) | (2) | (3) | (4) | (5) | (6) |
| --- | --- | --- | --- | --- | --- | --- |
|  | High General Health | Sick days per month | Smoker | Alcohol intake, days per week | Physically active, days per week | Walking > 10m, days per week |
| Impatience Z | -0.009 (0.009) | -0.001 (0.021) | 0.007 (0.007) | 0.015 (0.058) | -0.000 (0.036) | -0.071 (0.056) |
| Inc. impatience Z | -0.003 (0.009) | -0.018 (0.021) | 0.008 (0.007) | -0.107 (0.064) | -0.000 (0.036) | 0.065 (0.057) |
| Dec. impatience Z | 0.010 (0.013) | 0.013 (0.030) | -0.013 (0.010) | -0.177* (0.080) | 0.007 (0.052) | -0.055 (0.081) |
| Grit PoE | 0.036*** (0.008) | -0.002 (0.018) | 0.005 (0.006) | -0.046 (0.050) | 0.037 (0.031) | 0.125* (0.049) |
| Grit CoI | 0.028*** (0.007) | -0.031 (0.016) | 0.009 (0.006) | 0.023 (0.042) | -0.003 (0.028) | -0.001 (0.043) |
| Female | -0.035 (0.019) | 0.081 (0.044) | -0.041** (0.015) | -0.568*** (0.117) | -0.327*** (0.075) | 0.373** (0.117) |
| Age | -0.019*** (0.003) | 0.019* (0.008) | 0.011*** (0.003) | 0.011 (0.021) | -0.016 (0.014) | 0.010 (0.021) |
| Age squared | 0.000*** (0.000) | -0.000** (0.000) | -0.000*** (0.000) | 0.000 (0.000) | -0.000 (0.000) | -0.000 (0.000) |
| Married | -0.003 (0.022) | 0.105* (0.051) | -0.033 (0.018) | 0.059 (0.138) | -0.201* (0.087) | -0.057 (0.136) |
| Divorced | 0.034 (0.032) | 0.149* (0.075) | 0.084** (0.026) | -0.065 (0.196) | 0.066 (0.128) | -0.022 (0.200) |
| High education | 0.085*** (0.020) | -0.062 (0.046) | -0.078*** (0.016) | 0.252* (0.120) | -0.023 (0.079) | 0.162 (0.123) |
| No. of children | 0.009 (0.010) | -0.055* (0.022) | -0.017* (0.008) | -0.187** (0.062) | 0.038 (0.039) | -0.100 (0.060) |
| Home ownership | 0.024 (0.021) | -0.240*** (0.048) | -0.087*** (0.017) | 0.074 (0.134) | 0.237** (0.083) | 0.214 (0.129) |
| Log net income | 0.019 (0.016) | -0.109** (0.036) | -0.016 (0.012) | -0.103 (0.099) | 0.102 (0.062) | 0.020 (0.096) |
| Self-employed | 0.072 (0.041) | -0.148 (0.094) | -0.019 (0.033) | 0.317 (0.241) | 0.388* (0.162) | 0.025 (0.253) |
| *Adj. R^2^* | 0.099 | 0.036 | 0.056 | 0.131 | 0.051 | 0.008 |
| *N* | 2281 | 2279 | 2279 | 1398 | 2278 | 2278 |

Notes: OLS; standard errors in parentheses; High General Health is an indicator for an above median response; impatience measures are z-scores; CoI = Consistency of Interests Grit subscale, PoE = Perseverance of Effort Grit subscale; education is an indicator for an above median response; all models include a dummy for DI=0 for patient respondents waiting until the maximum delay of the choice list (see section 2); reduced sample sizes due to missing data for some dependent variables; */**/*** denote significance of difference from zero at 5% / 1% / 0.1%.

## Section C: Robustness to including extremely patient participants

Table C1: Summary statistics

|  | Impatience | DI-index | DQ-Grit Score | Grit Score | Grit PoE | Grit CoE |
| --- | --- | --- | --- | --- | --- | --- |
| Median | 0.632 | 0 | 4.875 | 2 | 1 | 0 |
| Mean | 2.718 | 0.525 | 4.895 | 2.423 | 1.518 | 0.905 |
| Std. dev. | 4.762 | 3.448 | 0.841 | 2.163 | 1.378 | 1.204 |
| N | 3421 | 2754 | 3370 | 3370 | 3370 | 3370 |

Notes: Impatience = annual discount rate as implied by first choice list with a 5-week up-front delay; Grit Score $\in$ [1, 7]; Grit Index $\in\{0, \ldots, 8\}$; Grit PoE and CoI Index $\in\{0, \ldots, 4\}$.

Table C2: Deviations from constant discounting

|  | Rohde 1 | Rohde 2 | Our data |
| --- | --- | --- | --- |
| Up-front delay | 0 weeks | 2 weeks | 5 weeks |
| Decreasing impatience (DI > 0) | 0.457 | 0.396 | 0.271 |
| Constant impatience (DI = 0) | 0.298 | 0.231 | 0.540 |
| Increasing impatience (DI < 0) | 0.245 | 0.374 | 0.190 |
| *N* | 94 | 91 | 2754 |

Notes: Shares of all participants reported; Rohde 1 and Rohde 2 are based on the two choice lists of the second experiment reported in Rohde (2019).

Table C3: Demographic correlates of time preference and grit

|  | (1) | (2) | (3) | (4) | (5) | (6) |
| --- | --- | --- | --- | --- | --- | --- |
|  | Impatience | Increasing Impatience | Decreasing Impatience | Grit Score | Grit CoI | Grit PoE |
| Female | -0.163 (0.186) | 0.003 (0.002) | 0.388* (0.163) | 0.189* (0.088) | 0.087 (0.056) | 0.103* (0.049) |
| Age | 0.085* (0.034) | 0.000 (0.000) | -0.013 (0.029) | 0.026 (0.016) | 0.017 (0.010) | 0.009 (0.009) |
| Age squared | -0.000 (0.000) | -0.000 (0.000) | 0.000 (0.000) | -0.000 (0.000) | -0.000 (0.000) | -0.000 (0.000) |
| Married | -0.046 (0.216) | -0.001 (0.002) | -0.386* (0.190) | 0.204* (0.102) | 0.098 (0.065) | 0.106 (0.057) |
| Divorced | 0.318 (0.318) | -0.005 (0.003) | -0.611* (0.280) | 0.125 (0.150) | 0.021 (0.095) | 0.103 (0.084) |
| High education | -0.660*** (0.195) | -0.002 (0.002) | -0.213 (0.170) | 0.210* (0.092) | 0.221*** (0.058) | -0.011 (0.051) |
| No. of children | 0.171 (0.096) | -0.000 (0.001) | 0.135 (0.084) | -0.005 (0.045) | -0.011 (0.029) | 0.006 (0.025) |
| Home ownership | -0.717*** (0.206) | -0.002 (0.002) | -0.259 (0.180) | -0.022 (0.096) | 0.068 (0.061) | -0.090 (0.054) |
| Log net income | -0.527*** (0.157) | 0.001 (0.001) | 0.157 (0.134) | 0.217** (0.074) | 0.067 (0.047) | 0.150*** (0.041) |
| Self-employed | 0.210 (0.398) | 0.002 (0.004) | 0.643 (0.350) | 0.471* (0.188) | 0.072 (0.119) | 0.399*** (0.105) |
| *Adj. R^2^* | 0.034 | 0.004 | 0.009 | 0.020 | 0.025 | 0.012 |
| *N* | 2903 | 2346 | 2346 | 2855 | 2855 | 2855 |

Notes: OLS with standard errors in parentheses; Increasing Impatience = absolute value of DI-index if negative; Decreasing Impatience = DI-index if positive; CoI = Consistency of Interests Grit subscale, PoE = Perseverance of Effort Grit subscale; Education is an indicator for above median education. */**/*** denote significance of difference from zero at 5% / 1% / 0.1%.

Table C4: Rank correlation coefficients

|  | Impatience | Increasing Impatience | Decreasing Impatience | Grit Score | Grit CoI |
| --- | --- | --- | --- | --- | --- |
| Inc. Impatience | -0.03 |  |  |  |  |
| Dec. Impatience | 0.19*** | -0.29*** |  |  |  |
| Grit Score | -0.05* | 0.02 | -0.02 |  |  |
| Grit CoI | -0.04* | 0.03 | -0.03 | 0.88*** |  |
| Grit PoE | -0.05* | 0.01* | 0.00 | 0.75*** | 0.39*** |

Notes: Spearman’s rank correlation coefficients; Impatience = discount rate as implied by first choice list with 5-week up-front delay; CoI = Consistency of Interests Grit subscale; PoE = Perseverance of Effort Grit subscale; */**/*** denote significance of difference from zero at 5% / 1% / 0.1%.

Table C5: Explaining Financial Dissatisfaction Score

|  | (1) | (2) | (3) | (4) | (5) |
| --- | --- | --- | --- | --- | --- |
| Impatience Z | 0.098*** (0.026) | 0.067* (0.029) | 0.095*** (0.025) | 0.069* (0.028) | 0.069* (0.028) |
| Inc. impatience Z | 0.077** (0.027) | 0.040 (0.030) | 0.072** (0.026) | 0.041 (0.030) | 0.041 (0.030) |
| Dec. impatience Z | -0.033 (0.041) | -0.027 (0.043) | -0.036 (0.040) | -0.027 (0.042) | -0.033 (0.042) |
| Grit Score |  |  | -0.151*** (0.012) | -0.107*** (0.013) |  |
| Grit PoE |  |  |  |  | -0.000 (0.026) |
| Grit CoI |  |  |  |  | -0.197*** (0.023) |
| Female |  | 0.001 (0.065) |  | 0.010 (0.064) | 0.001 (0.064) |
| Age |  | 0.004 (0.012) |  | 0.009 (0.012) | 0.011 (0.012) |
| Age squared |  | -0.000* (0.000) |  | -0.000* (0.000) | -0.000* (0.000) |
| Married |  | -0.094 (0.074) |  | -0.069 (0.072) | -0.068 (0.072) |
| Divorced |  | -0.029 (0.105) |  | -0.006 (0.104) | -0.009 (0.103) |
| High education |  | -0.134* (0.067) |  | -0.118 (0.066) | -0.095 (0.066) |
| No. of children |  | 0.056 (0.034) |  | 0.052 (0.034) | 0.049 (0.034) |
| Home ownership |  | -0.169* (0.072) |  | -0.186** (0.071) | -0.177* (0.070) |
| Log net income |  | 0.001 (0.059) |  | 0.020 (0.058) | 0.009 (0.058) |
| Self-employed |  | 0.015 (0.136) |  | 0.066 (0.134) | 0.029 (0.134) |
| Ladder of life |  | -0.099*** (0.025) |  | -0.081** (0.025) | -0.074** (0.025) |
| High Ease of Living |  | -0.573*** (0.066) |  | -0.549*** (0.065) | -0.543*** (0.065) |
| *Adj. R^2^* | 0.007 | 0.151 | 0.060 | 0.178 | 0.187 |
| *N* | 2713 | 1982 | 2712 | 1981 | 1981 |

Notes: OLS; standard errors in parentheses; dependent variable is the financial dissatisfaction score; impatience measures are z-scores; CoI = Consistency of Interests Grit subscale, PoE = Perseverance of Effort Grit subscale; education and ease of living are indicators for above median scores; reduced sample sizes due to missing variables for some participants; */**/*** denote significance of difference from zero at 5% / 1% / 0.1%.

Table C6: Explaining Health Dissatisfaction Score

|  | (1) | (2) | (3) | (4) | (5) |
| --- | --- | --- | --- | --- | --- |
| Impatience Z | -0.020 (0.026) | -0.015 (0.036) | -0.022 (0.025) | -0.016 (0.036) | -0.017 (0.036) |
| Inc. impatience Z | 0.020 (0.027) | -0.014 (0.041) | 0.017 (0.026) | -0.014 (0.040) | -0.015 (0.040) |
| Dec. impatience Z | -0.017 (0.041) | 0.029 (0.051) | -0.019 (0.041) | 0.022 (0.051) | 0.014 (0.051) |
| Grit Score |  |  | -0.086*** (0.012) | -0.056*** (0.016) |  |
| Grit PoE |  |  |  |  | 0.036 (0.032) |
| Grit CoI |  |  |  |  | -0.130*** (0.027) |
| Female |  | -0.184* (0.076) |  | -0.178* (0.076) | -0.176* (0.076) |
| Age |  | 0.007 (0.014) |  | 0.010 (0.014) | 0.010 (0.014) |
| Age squared |  | -0.000 (0.000) |  | -0.000 (0.000) | -0.000 (0.000) |
| Married |  | -0.031 (0.089) |  | -0.017 (0.089) | -0.017 (0.088) |
| Divorced |  | 0.146 (0.127) |  | 0.155 (0.127) | 0.153 (0.126) |
| High education |  | -0.045 (0.078) |  | -0.033 (0.078) | -0.017 (0.078) |
| No. of children |  | -0.023 (0.041) |  | -0.024 (0.040) | -0.027 (0.040) |
| Home ownership |  | -0.077 (0.087) |  | -0.083 (0.087) | -0.063 (0.087) |
| Log net income |  | 0.200** (0.064) |  | 0.211*** (0.064) | 0.205** (0.063) |
| Self-employed |  | 0.022 (0.156) |  | 0.046 (0.155) | 0.017 (0.155) |
| High general health |  | -0.648*** (0.081) |  | -0.601*** (0.082) | -0.611*** (0.082) |
| Sick days |  | 0.112** (0.042) |  | 0.107** (0.042) | 0.100* (0.041) |
| Smoking |  | 0.301** (0.105) |  | 0.333** (0.104) | 0.331** (0.104) |
| Alcohol days |  | -0.014 (0.018) |  | -0.015 (0.017) | -0.013 (0.017) |
| Activity days |  | -0.109*** (0.021) |  | -0.109*** (0.021) | -0.110*** (0.021) |
| Walking days |  | -0.050*** (0.014) |  | -0.049*** (0.014) | -0.051*** (0.014) |
| *Adj. R^2^* | -0.001 | 0.117 | 0.017 | 0.124 | 0.130 |
| *N* | 2712 | 1397 | 2712 | 1397 | 1397 |

Notes: OLS; standard errors in parentheses; dependent variable is the health dissatisfaction score; impatience measures are z-scores; CoI = Consistency of Interests Grit subscale, PoE = Perseverance of Effort Grit subscale; education and general health are indicators for above median scores; reduced sample sizes due to missing variables for some participants; */**/*** denote significance of difference from zero at 5% / 1% / 0.1%.

Table C7: Explaining financial outcomes

|  | (1) | (2) | (3) | (4) | (5) | (6) | (7) |
| --- | --- | --- | --- | --- | --- | --- | --- |
|  | Balance on Bank Accounts | Sum of Investments | Log Net Inc. | High Fin. Satis. | Ladder | High Ease of Living | Home Ownership |
| Impatience Z | -8.463*** (2.400) | -7.624 (12.495) | -0.077*** (0.012) | -0.044*** (0.009) | -0.117*** (0.025) | -0.054*** (0.010) | -0.031*** (0.008) |
| Inc. impatience Z | -1.925 (2.425) | -3.492 (16.234) | -0.005 (0.013) | -0.020* (0.010) | -0.057* (0.026) | -0.027** (0.010) | -0.013 (0.008) |
| Dec. impatience Z | -1.182 (3.042) | -33.220 (34.712) | 0.022 (0.019) | -0.028 (0.015) | 0.007 (0.040) | -0.027 (0.015) | -0.010 (0.013) |
| Grit PoE | -1.929 (1.834) | 9.324 (7.302) | 0.028* (0.011) | 0.007 (0.009) | -0.009 (0.024) | 0.004 (0.009) | -0.018* (0.008) |
| Grit CoI | -0.290 (1.605) | 1.265 (6.134) | 0.017 (0.010) | 0.048*** (0.008) | 0.138*** (0.021) | 0.037*** (0.008) | 0.022** (0.007) |
| Female | -7.263 (4.191) | -14.478 (17.174) | -0.462*** (0.026) | -0.009 (0.019) | -0.004 (0.052) | -0.061** (0.021) | -0.040* (0.017) |
| Age | 1.972** (0.668) | 5.245 (2.686) | 0.061*** (0.004) | 0.004 (0.003) | 0.004 (0.008) | 0.001 (0.004) | 0.013*** (0.003) |
| Age squared | -0.011 (0.007) | -0.041 (0.025) | -0.001*** (0.000) | -0.000 (0.000) | -0.000 (0.000) | -0.000 (0.000) | -0.000*** (0.000) |
| *Adj. R^2^* | 0.052 | 0.015 | 0.211 | 0.048 | 0.033 | 0.035 | 0.020 |
| *N* | 1138 | 254 | 2313 | 2399 | 2480 | 2244 | 2708 |

Notes: OLS; standard errors in parentheses; dependent variables Balance on Bank Accounts and Sum of Investments in thousand euros; impatience measures are z-scores; CoI = Consistency of Interests Grit subscale, PoE = Perseverance of Effort Grit subscale; Grit and education variables are indicators for above median scores; Grit variables are indicators for above median scores; reduced sample sizes due to missing data for some dependent variables; */**/*** denote significance of difference from zero at 5% / 1% / 0.1%.

Table C8: Explaining health outcomes

|  | (1) | (2) | (3) | (4) | (5) | (6) |
| --- | --- | --- | --- | --- | --- | --- |
|  | High General Health | Sick days per month | Smoking | Alcohol intake, days per week | Physically active, days per week | Walking > 10m, days per week |
| Impatience Z | -0.022** (0.008) | 0.019 (0.018) | 0.017** (0.006) | 0.007 (0.050) | -0.016 (0.031) | -0.113* (0.048) |
| Inc. impatience Z | -0.007 (0.008) | 0.002 (0.019) | 0.010 (0.007) | -0.129* (0.058) | 0.017 (0.032) | 0.012 (0.050) |
| Dec. impatience Z | 0.010 (0.012) | 0.001 (0.029) | -0.011 (0.010) | -0.115 (0.074) | 0.021 (0.050) | -0.102 (0.077) |
| Grit PoE | 0.035*** (0.007) | -0.007 (0.017) | 0.007 (0.006) | -0.055 (0.046) | 0.044 (0.029) | 0.140** (0.045) |
| Grit CoI | 0.031*** (0.006) | -0.043** (0.015) | 0.002 (0.005) | 0.052 (0.039) | -0.011 (0.026) | 0.010 (0.040) |
| Female | -0.050** (0.016) | 0.137*** (0.038) | -0.027* (0.013) | -0.506*** (0.100) | -0.403*** (0.065) | 0.261** (0.100) |
| Age | -0.016*** (0.002) | 0.003 (0.006) | 0.005** (0.002) | 0.004 (0.016) | -0.006 (0.010) | 0.014 (0.015) |
| Age squared | 0.000*** (0.000) | -0.000 (0.000) | -0.000** (0.000) | 0.000* (0.000) | -0.000 (0.000) | -0.000 (0.000) |
| *Adj. R^2^* | 0.095 | 0.010 | 0.007 | 0.135 | 0.045 | 0.008 |
| *N* | 2680 | 2678 | 2678 | 1583 | 2677 | 2677 |

Notes: OLS; standard errors in parentheses; High General Health is an indicator for an above median response; impatience measures are z-scores; CoI = Consistency of Interests Grit subscale, PoE = Perseverance of Effort Grit subscale; reduced sample sizes due to missing data for some dependent variables; */**/*** denote significance of difference from zero at 5% / 1% / 0.1%.
